# PCSK9 inhibitor treatment and outcomes in patients with atherosclerotic cardiovascular disease but without prior ischaemic events: an observational study

**DOI:** 10.1093/eurheartj/ehag176

**Published:** 2026-04-16

**Authors:** Deepak L Bhatt, Pasquale Perrone Filardi, Irfan Khan, José Tuñón, Nikolaus Marx, Manuel Castro Cabezas, Katherine Andrade, Alexandra Koumas, Zhouyang Lou, Lale Tokgözoğlu

**Affiliations:** Mount Sinai Fuster Heart Hospital, Icahn School of Medicine at Mount Sinai, 1 Gustave L. Levy Place, NewYork, NY 10029, USA; Department of Advanced Biomedical Sciences, Federico II University of Naples, Italy; Integrated Evidence Generation, Sanofi, Morristown, NJ, USA; Department of Cardiology, Fundación Jiménez Díaz, Autónoma University, CIBERCV, Madrid, Spain; Department of Internal Medicine I, Cardiology, RWTH Aachen University, Aachen, Germany; Department of Internal Medicine, Endocrinology, Diabetes and Vascular Medicine, Franciscus Gasthuis & Vlietland, Rotterdam; Erasmus MC, Rotterdam, The Netherlands; Optum Life Sciences, Optum, Eden Prairie, MN, USA; Axtria, Berkeley Heights, NJ, USA; Axtria, Berkeley Heights, NJ, USA; Department of Cardiology, Hacettepe University, Ankara, Turkey

**Keywords:** Atherosclerotic cardiovascular disease, PCSK9 inhibitor

## Abstract

**Background and Aims:**

Low-density lipoprotein cholesterol (LDL-C)-lowering therapies are proven effective in atherosclerotic cardiovascular disease (ASCVD), but real-world evidence for proprotein convertase subtilisin/kexin type 9 inhibitor (PCSK9i) monoclonal antibodies (mAb) remains limited. This study evaluated their impact in patients with ASCVD without prior ischaemic events.

**Methods:**

Patients initiating PCSK9i mAb from January 2016 to December 2022 were identified in the Optum Research Database. A 1:2 propensity score-matched comparator cohort of PCSK9i non-initiators was developed. The primary endpoint was a composite of non-fatal myocardial infarction, non-fatal ischaemic stroke, or all-cause mortality. Key outcomes from the parametric G-formula were 5-year event rates, relative risk reduction (RRR) and absolute risk reduction (ARR), with intention-to-treat (ITT) analysis. Additional outcomes for PCSK9i mAb initiators included absolute and percent LDL-C reduction from baseline.

**Results:**

Overall, 19 670 patients met selection criteria (6545 PCSK9i mAb initiators; 13 125 non-initiators). Baseline characteristics were well-balanced. Under ITT, estimated 5-year event rates were 17.5% [95% confidence interval (CI) 15.5%, 19.5%] with PCSK9i mAb vs 25.4% (95% CI 23.6%, 27.1%) without PCSK9i, yielding a RRR of 30.9% and ARR of 7.8%. Individual endpoints showed RRRs of 28.3% for myocardial infarction (*P* < .0001), 26.4% for ischaemic stroke (*P* = .02), and 28.5% for all-cause mortality (*P* < .0001). Among initiators, mean baseline and follow-up LDL-C were 117.8 and 54.7 mg/dL (on-treatment analysis), respectively, representing an absolute reduction of 63.1 mg/dL and percent reduction of 53.6%.

**Conclusions:**

In ASCVD patients without prior events in clinical practice, PCSK9i mAb treatment was associated with lower ischaemic event and mortality rates.

## Structured Graphical Abstract

**Structured Graphical Abstract:**
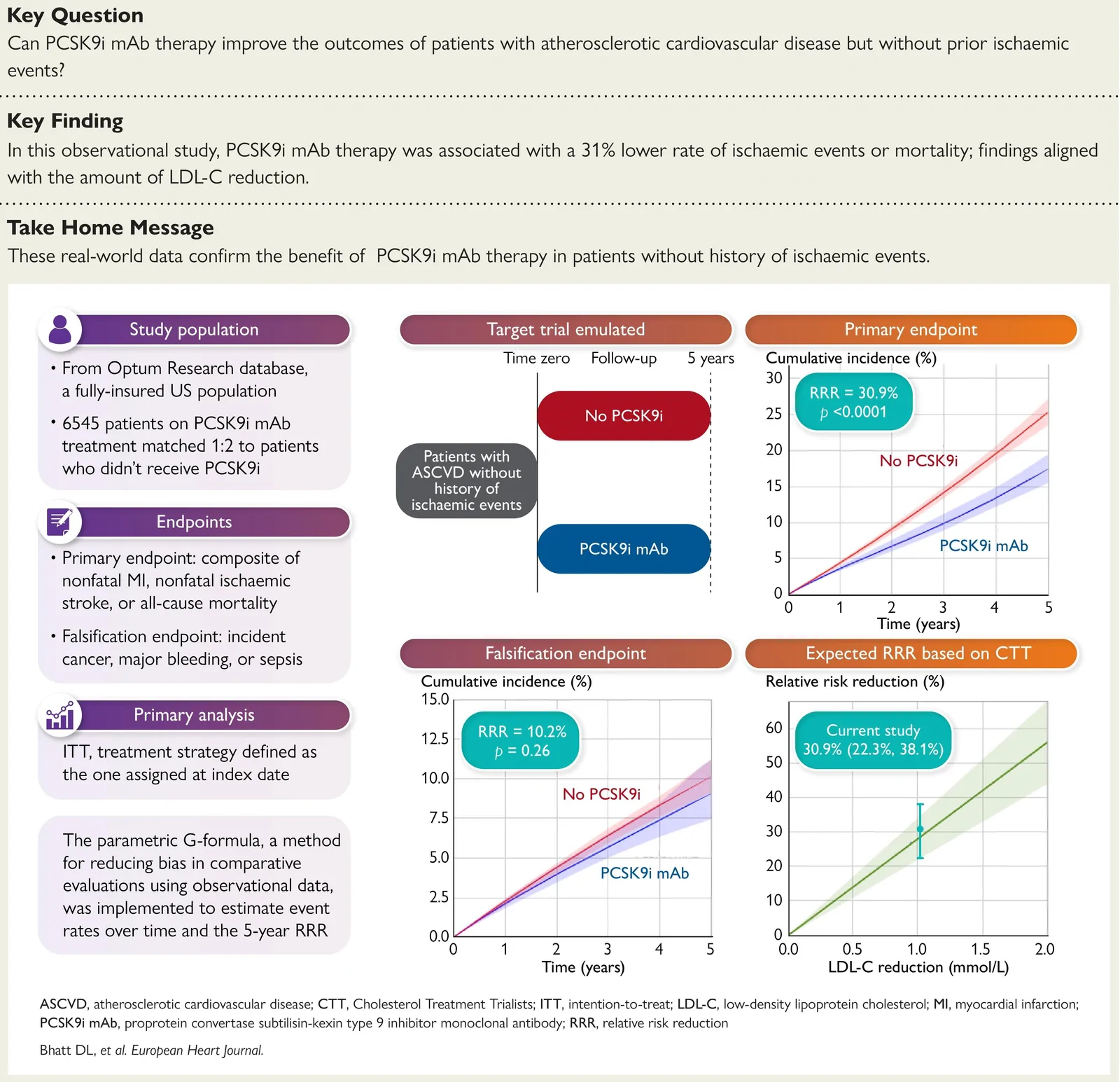

**See the editorial comment for this article ‘PCSK9 inhibition in patients without previous cardiovascular events but at high risk: the earlier the better’, by D. Sinning and U. Landmesser, https://doi.org/10.1093/eurheartj/ehag456.**

## Introduction

Low-density-lipoprotein cholesterol (LDL-C) reduction has been proven in randomized controlled trials (RCTs) to reduce ischaemic events.^1–4^ Additional evidence, such as from Mendelian randomization and epidemiologic studies, also supports reduction in adverse cardiovascular outcomes with lower LDL-C levels.^5,6^ The Cholesterol Treatment Trialists’ (CTT) Collaboration convincingly demonstrated that every ∼40 mg/dL (1 mmol/L) reduction in LDL-C is associated with a 22% reduction in major adverse cardiovascular events (MACE).^7^

Proprotein convertase subtilisin/kexin type 9 inhibitor (PCSK9i) monoclonal antibodies (mAb) have been demonstrated in RCTs to lower the rate of MACE by approximately 20%.^1,2^ In high-risk atherosclerotic cardiovascular disease (ASCVD) patients such as those with acute coronary syndromes (ACS), rates of all-cause mortality were also lower.^3^ However, there are limited data evaluating their effectiveness in non-trial populations (see Supplementary data online, *eMethods S1*), for periods of observation longer than in RCTs, and in populations with lower rates of background statin therapy than mandated in the PCSK9i mAb trials.

To address these gaps, the OPTIMUS programme (Outcomes and PrevenTIon Programme with PCSK9i Monoclonal Antibodies in ASCVD PopUlation without Prior ISchaemic Events) was developed. Within this programme, using contemporary methods on comparative evaluation,^8,9^ the current OPTIMUS–CLASS study evaluated the association between use of the PCSK9i mAb class (alirocumab and evolocumab) vs no PCSK9i on 5-year MACE and all-cause mortality in a large and contemporary population representing usual clinical practice in the USA among patients with clinically documented ASCVD and no prior ischaemic events.

## Methods

### Source data

Patients were identified in the Optum Research Database (ORD), a large de-identified US research database that contains administrative claims from commercial and Medicare Advantage plans, containing both medical and pharmacy information. Data on mortality were also included, which are captured from a variety of sources in order to provide the most complete information on death available, including the Social Security Administration’s Death Master File (SSA-DMF). Elements used for this study included demographics, diagnoses, procedures, prescription fills, and lipid measurements, enabling robust time-to-event analyses with clear censoring information and reliable ascertainment of major cardiovascular and mortality outcomes. The database, methodology for identification of patients, and identification of endpoints have been described previously.^10^ The study was reviewed by the WCG Institutional Review Board (IRB) and granted IRB exemption under 45 § CFR 46.104(d)(4); informed consent was therefore not required. The underlying data contains proprietary elements that cannot be broadly disclosed or made publicly available. However, these data can be made available to a third party via the corresponding author upon reasonable request, assuming data security and privacy protocols are in place, and that the third party has executed the standard licence agreements.

### Study population

From January 2016 to December 2022, patients initiating PCSK9i mAb (specifically alirocumab or evolocumab) were identified, with the index date defined as the start of treatment. Inclusion was restricted to individuals with documented ASCVD, characterized by atherosclerotic involvement of coronary, cerebrovascular, or peripheral arteries. Patients with a recorded history of ischaemic events, including myocardial infarction (MI), unstable angina (UA), or ischaemic stroke, concurrent with or preceding their earliest ASCVD diagnosis, were excluded. Ischaemic history was determined by relevant diagnostic codes during the 12-month baseline period prior to index. To minimize misclassification, history of these events was assessed across all available information before index (not limited to the 12-month baseline), using diagnostic codes that captured both incident events and codes indicating a history of acute ischaemic events. A baseline LDL-C measurement within the 12-month pre-index period was also required (see Supplementary data online, *eTable S1* and *eMethods S2*). For the primary analysis, a recorded LDL-C measurement within the baseline period was also required. Additional inclusion criteria are detailed in Supplementary data online, *eTable S1* and *eMethods S2*. Definitions for ASCVD and ischaemic events were aligned with those used in a previously published study.^10^

A comparator population which did not initiate a PCSK9i [either a mAb or small interfering RNA (siRNA)] was developed as follows. Patients with any evidence of ASCVD were initially selected. Patients with evidence of ischaemic events either concurrent with or prior to the earliest record of ASCVD were excluded. Any point after record of ASCVD and before a potential ischaemic event was eligible to be assigned as index (time zero). This index represented a point where the patient met all the inclusion criteria in a hypothetical trial enrolling this population who would qualify for therapy. To make a pragmatic and valid selection of a set of patients and index dates from a large available pool, index dates were randomly selected per the schematic in Supplementary data online, *eFigure S1*, with additional confirmation on whether the eligibility criteria were still met at the index selected. The resulting comparator population was further narrowed by requiring baseline LDL-C measurement and 1:2 propensity score matching (PSM) with those initiating PCSK9i mAb. Further details of the matching are provided in Supplementary data online, *eTable S2*.

An expanded population that also included patients with missing baseline LDL-C was developed for supplementary analyses. A separate propensity score matching was undertaken in the group with missing baseline LDL-C. The groups (those with missing and non-missing baseline LDL-C) were then pooled, and baseline LDL-C was imputed in those with missing LDL-C. See supplement for further details. Additionally, we undertook a subgroup analysis comparing patients who were receiving statin therapy at baseline vs those who were not.

### Baseline characteristics

Baseline characteristics that were assessed were demographics, ASCVD related conditions, other comorbidities, lipid-lowering therapy (LLT) use, other cardiovascular standard of care medications, and LDL-C.

### Endpoints and estimands

The population was followed over time for the occurrence of the primary endpoint, defined as a composite representing first occurrence of non-fatal MI, non-fatal ischaemic stroke, or all-cause mortality. Cardiovascular death was not included because timely linkage to the National Death Index was unavailable; all-cause mortality was selected to minimize misclassification. Ischaemic events (non-fatal MI and non-fatal ischaemic stroke) required hospitalization to enhance specificity.^10^ All-cause mortality was captured from a variety of sources in order to provide the most complete information on death available, including the Social Security Administration’s Death Master File (SSA-DMF), an approach validated in prior large-scale cardiovascular effectiveness studies and shown to yield findings concordant with European registries.^11^ In addition to the composite, the individual endpoints of non-fatal MI, non-fatal ischaemic stroke, and all-cause mortality were also evaluated.

Study estimands were the relative risk reduction (RRR) and absolute risk reduction (ARR) over 5 years with an intention-to-treat (ITT) analysis, meaning the intervention is assigned at index. If Eand $E_{0}$ denote the event rates at 5-years for the treated and comparator arms, RRR was equal to $(E_{0}-E)/E_{0}$, and ARR was equal to $E_{0}-E$.

### Falsification endpoint

Patients were also followed for a falsification endpoint, defined as composite of incident cancer, hospitalized major bleeding, or hospitalized sepsis. The falsification endpoint, also referred to as a negative control outcome,^12^ is an outcome that is not expected to be influenced by the treatment under consideration. Findings of a null effect on a falsification endpoint can help enhance the validity of findings for the primary endpoint. Incident cancer was identified as follows: at least two non-diagnostic claims at least 30 days apart with a code for the same type of cancer post-index or with evidence of anti-cancer system therapy after diagnosis, and with no evidence of cancer diagnosis or treatment for cancer during baseline. Hospitalized major bleeding or sepsis were identified via presence of relevant codes together with evidence of an inpatient hospitalization. To minimize contamination by cardiovascular morbidity status, falsification follow-up was censored at the earliest of non-fatal MI, non-fatal ischaemic stroke, death, hospitalization for unstable angina, or any coronary or peripheral revascularization.

### Survival analysis via the parametric G-formula

As described previously, the study population was developed via PSM with a 1:2 ratio for PCSK9i mAb initiators and PCSK9i non-initiators. To ensure optimal bias reduction, the parametric G-formula was subsequently used to estimate the event rate curves over time for the primary endpoint, which may be interpreted as the expected event rates under assignment of PCSK9i mAb and no PCSK9i, under identifiability assumptions as detailed in the supplement. A thorough explanation of this methodology is available in Hernán and Robins and other technical references,^8,9^ and supplement provides additional description. Given the sequential application of PSM and the parametric G-formula, the resulting estimates meet the criteria for double robustness—whereby the parametric G-formula provides additional bias reduction independent of the propensity score model.

The estimates of event rates over time, RRR, and ARR, obtained from the parametric G-formula may be considered as unbiased under the identifiability assumptions, as summarized in Supplementary data online, *eMethods S3*. The key identifiability assumption is that of conditional exchangeability, meaning the available confounder set is sufficient to adjust for the baseline differences between the PCSK9i mAb and no PCSK9i groups. The confounder set in this study represented all baseline characteristics. The parametric G-formula utilized the discrete-time survival analysis framework,^13^ with censoring due to end of enrolment in the health plan or end of study period.

### Reduction in LDL-C and concordance with CTT data

The absolute and percent reductions in LDL-C from initiating PCSK9i mAb were calculated. This analysis was undertaken in population initiating PCSK9i mAb with available LDL-C at baseline and follow-up. Full details of the estimation are available in Supplementary data online, *eMethods S4*. The estimated RRR from the present study was compared with the expected RRR from CTT data^7^ as the absolute LDL-C reduction in mmol/L multiplied by 28% [95% confidence interval (CI) 22%, 34%]. This value was used instead of the more commonly cited figure of 22% (95% CI 20%, 24%) because it represents a secondary prevention population with more vs less intensive therapy as comparison, which is closer to the setting for the current study. The CTT meta-analysis defined its primary endpoint as a composite of coronary heart disease death, non-fatal MI, fatal or non-fatal stroke, or coronary revascularization, which differs from our 3-component composite.

## Results

### Baseline characteristics

A total of 19 670 patients met the selection criteria, with 6545 PCSK9i mAb initiators and 13 125 PCSK9i non-initiators (1:2 ratio). *Table 1* summarizes the baseline characteristics, which were generally well-balanced across the two groups. A total of 50.7% of patients were male with a mean [standard deviation (SD)] age of 68.1 (10.2) years. Of the 19 670 patients, 73.8% had stable angina, 16.5% had received percutaneous coronary intervention (PCI), and 11.9% had received coronary artery bypass grafting (CABG). Ischaemic cerebrovascular disease was present in 9.6%, 4.0% had peripheral artery disease of the lower extremities, and 4.9% had atherosclerosis of the aorta. Other comorbidities included diabetes mellitus (35.9%), hypertension (79.5%), heart failure (8.6%), and chronic kidney disease stages III–V (10.4%). ASCVD types frequently overlapped (e.g. a patient could have stable angina plus prior PCI or CABG, or both coronary and peripheral artery disease), reflecting the multisite and polyvascular nature of disease. Consequently, the sum of percentages may exceed 100%.

**Table 1:** Baseline characteristics of the study population

	PCSK9i mAb (*n* = 6545)	No PCSK9i (*n* = 13 125)	Study population (*n* = 19 670)
Demographics, %			
Age, years			
Mean (SD)	67.9 (9.1)	68.2 (10.6)	68.1 (10.2)
18–44	1.3	1.7	1.6
45–64	30.7	30.8	30.8
≥65	68.0	67.5	67.6
Male sex	50.8	50.7	50.7
Insurance			
Medicare advantage^a^	68.1	69.8	69.3
Commercial	31.9	30.2	30.7
ASCVD characteristics, %			
Coronary conditions	76.7	76.0	76.1
Stable angina	74.7	73.5	73.8
Other IHD	17.3	17.2	17.2
Prior revascularization	25.6	25.5	25.5
PCI	16.7	16.4	16.5
CABG	12.0	11.9	11.9
Ischaemic cerebrovascular disease	9.4	9.6	9.6
Peripheral artery disease	19.1	19.4	19.3
Lower extremities	3.9	4.1	4.0
Aorta	4.9	4.9	4.9
Other^b^	10.3	10.4	10.4
Other comorbidities, %			
Diabetes	35.9	35.8	35.9
No insulin	23.9	23.8	23.9
On insulin	12.0	12.0	12.0
Hypertension	79.5	79.5	79.5
Liver disease	5.1	5.2	5.2
COPD	9.9	9.9	9.9
Heart failure	8.7	8.5	8.6
Chronic kidney disease	10.7	10.4	10.4
Stage III	9.2	8.9	8.9
Stage IV^c^	1.5	1.5	1.5
Atrial fibrillation	9.7	9.7	9.7
Tobacco use	17.6	17.7	17.6
Cancer	11.9	11.3	11.4
Diagnosis	8.9	9.0	8.9
Medication	4.8	4.6	4.6
LLT use during 1-year prior to index, %			
Any statin use	61.0	61.6	61.4
Low-intensity	5.5	6.5	6.2
Moderate-intensity	23.8	25.9	25.3
High-intensity	31.7	29.3	29.9
Ezetimibe	33.4	33.3	33.3
Ezetimibe without statin	11.3	11.0	11.1
Icosapent ethyl	3.1	3.0	3.0
Bempedoic acid	0.4	0.5	0.5
Any statin, ezetimibe or bempedoic acid	72.4	72.7	72.6
Other CV SOC during 1-year prior to index, %			
Beta-blockers	52.6	52.3	52.4
ACEi/ARB	58.2	58.1	58.1
Antiplatelets^d^	23.8	23.5	23.6
Anticoagulants	10.5	10.5	10.5
LDL-C measures			
Mean (SD), mmol/L	3.0 (1.1)	3.0 (1.1)	3.0 (1.1)
Mean (SD), mg/dL	117.8 (42.0)	116.1 (43.2)	116.5 (42.9)
Median, mmol/L	3.1	2.9	2.9
Median, mg/dL	118.0	112.0	113.0

ACEi, angiotensin-converting-enzyme inhibitor; ARB, angiotensin receptor blocker; ASCVD, atherosclerotic cardiovascular disease; CABG, coronary artery bypass graft; COPD, chronic obstructive pulmonary disease; CV, cardiovascular; IHD, ischaemic heart disease; LDL-C, low-density lipoprotein cholesterol; LLT, lipid-lowering therapy; mAb, monoclonal antibody; PCI, percutaneous coronary intervention; PCSK9i, proprotein convertase subtilisin/kexin type 9 inhibitor; SD, standard deviation; SOC, standard of care.

^a^Represents health plan in the US offered by a private company that contracts with Medicare to provide patients with hospital and medical insurance benefits.

^b^Includes any type of disease that is non-coronary, non-cerebrovascular, or does not involve the lower extremities or aorta.

^c^Includes dialysis or renal transplant.

^d^Includes clopidogrel, ticagrelor, and prasugrel; 98.8% use in this category was for these agents. Additional agents were anagrelide, cilostazol, dipyridamole (including aspirin combo), pentoxifylline, and vorapaxar sulphate.

Percentages for ASCVD characteristics may exceed 100% because many patients have disease in multiple vascular beds.

Medication data in *Table 1* represents prescribed medications with filled prescriptions. In the study population, 61.4% received a statin, with 29.9% receiving a high-intensity statin. Ezetimibe use was 33.3%, while ezetimibe use without a statin was 11.1%; 27.4% were not receiving any LLT, including statins, ezetimibe, or bempedoic acid. P2Y_12_ inhibitors were utilized in 23.6%. Baseline LDL-C had a median of 113.0 mg/dL and a mean of 116.5 mg/dL. For patients initiating PCSK9i mAb, the median was 118.0 mg/dL.

Baseline characteristics of the expanded population evaluated are available in Supplementary data online, *eTable S3*.

### Event rates for the primary endpoint, relative and absolute risk reduction

**Figure 1:**
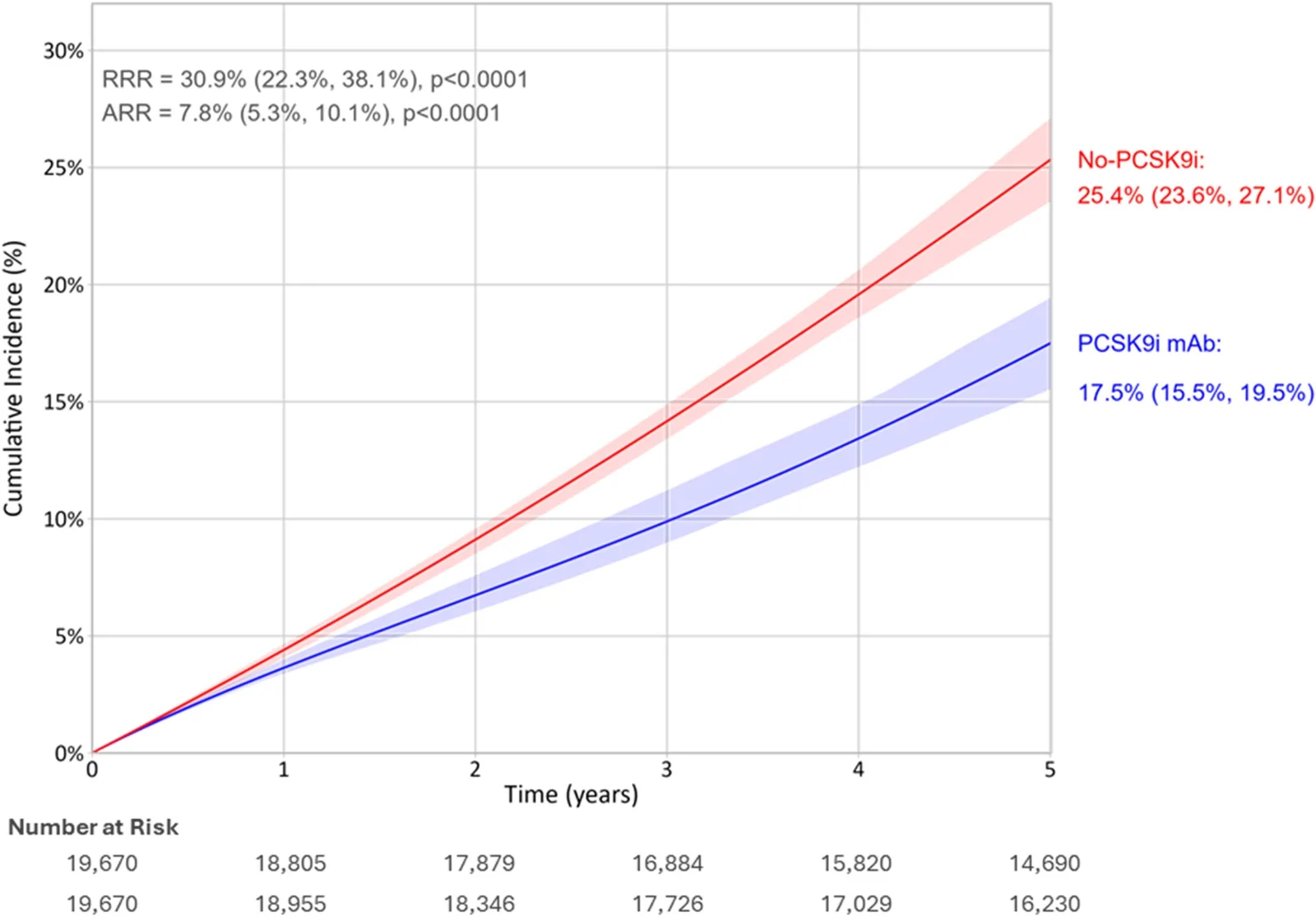
Primary composite endpoint for the study population—intention-to-treat analysis. ARR, absolute risk reduction; mAb, monoclonal antibody; PCSK9i, proprotein convertase subtilisin/kexin type 9 inhibitor; RRR, relative risk reduction. Numbers in parenthesis represent the 95% confidence intervals

The estimated event rates over time for the primary endpoint for the PCSK9i mAb and no PCSK9i groups with the parametric G-formula are summarized in *Figure 1*. For the ITT analysis without PCSK9i, the 5-year event rate was 25.4% (95% CI 23.6%, 27.1%) and with PCSK9i mAb it was 17.5% (95% CI 15.5%, 19.5%). This represented an RRR of 30.9% (95% CI 22.3%, 38.1%) and an ARR of 7.8% (95% CI 5.3%, 10.1%). Estimated event rates and RRR at 5 years for individual endpoints included in the composite primary endpoint are reported in Supplementary data online, *eTable S4*. Supplementary data online, *eFigure S2* shows the unadjusted event rates over time for the primary endpoint for the overall population (i.e. before application of the parametric G-formula). The primary endpoint result for the expanded population is available in Supplementary data online, *eFigure S3*. Patients receiving statin therapy at baseline had an RRR of 25.7%, compared with 34.8% among those not on statins (see Supplementary data online, *eFigure S4*). Both reductions were statistically significant (*P* < .0001), but the magnitude of the RRR difference between the statin and no statin groups was not (*P* = .42).

Individual endpoint analyses (see Supplementary data online, *eTable S4*) showed the following 5-year results. For non-fatal MI, event rates were 5.2% (95% CI 4.3%, 6.3%) with PCSK9i mAb vs 7.3% (95% CI 6.6%, 8.1%) without, yielding an RRR of 28.3% (95% CI 12.0%, 40.6%; *P* < .0001) and an ARR of 2.1% (95% CI 0.8%, 3.1%; *P* < .0001). For non-fatal ischaemic stroke, rates were 4.0% (95% CI 3.1%, 4.7%) vs 5.4% (95% CI 4.6%, 6.3%), corresponding to an RRR of 26.4% (95% CI 6.5%, 45.7%; *P* = .02) and an ARR of 1.5% (95% CI 0.3%, 2.7%; *P* = .02). For all-cause mortality, rates were 8.7% (95% CI 7.5%, 9.8%) vs 12.2% (95% CI 11.2%, 13.0%), representing an RRR of 28.5% (95% CI 16.6%, 39.1%; *P* < .0001) and an ARR of 3.5% (95% CI 2.0%, 4.9%; *P* < .0001).

### Reduction in LDL-C and comparison with CTT data

**Figure 2:**
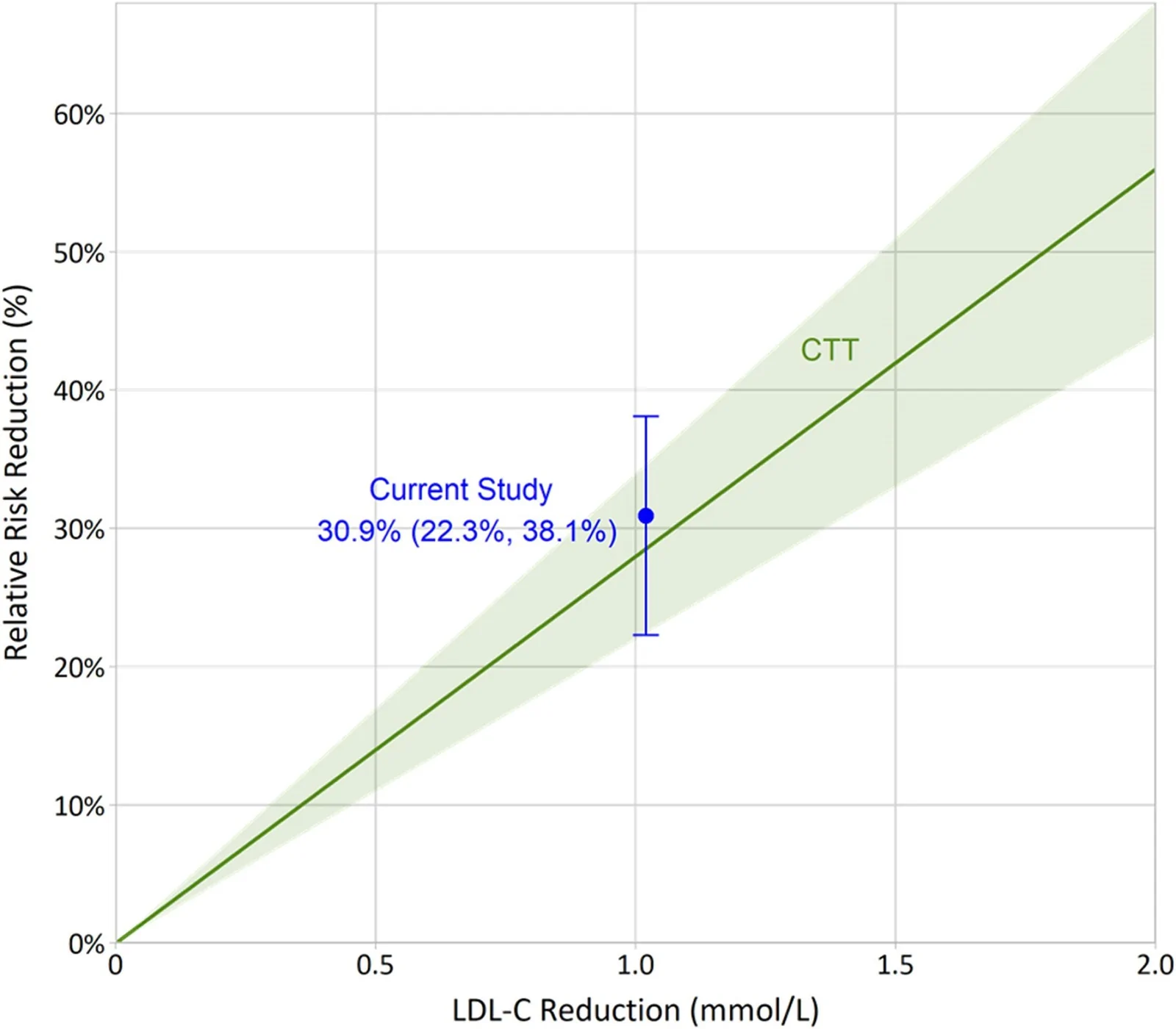
Comparison of findings with Cholesterol Treatment Trialists’ data. CTT, Cholesterol Treatment Trialists; ITT, intention to treat; LDL-C, low-density lipoprotein cholesterol. Numbers in parenthesis represent the 95% confidence intervals. CTT relative risk reduction 28.8% (95% CI 22.6%, 35.0%) per mmol/L reduction in LDL-C. Our composite is a 3-component endpoint (non-fatal myocardial infarction, non-fatal ischaemic stroke, all-cause mortality) and differs from the 5-component CTT major vascular event definition

In patients initiating PCSK9i mAb with LDL-C measurement at baseline and follow-up, the mean baseline LDL-C was 117.8 mg/dL, and the mean follow-up LDL-C for ITT analysis was 78.0 mg/dL, representing an absolute reduction of 39.8 mg/dL (1.0 mmol/L) and a percent reduction of 33.8%. The expected RRR from the CTT meta-analysis was therefore 28.8% (95% CI 22.6%, 35.0%). *Figure 2* presents the expected RRRs vs absolute reduction in LDL-C from CTT as a continuous line, with an overlay of the RRRs estimated from our study. The CI for the ITT estimate of RRR overlapped with the expected range from CTT data. With per-protocol analysis, mean follow-up LDL-C was 54.7 mg/dL (1.4 mmol/L), corresponding to an absolute reduction of 63.1 mg/dL (1.6 mmol/L) and a percentage reduction of 53.6% (see Supplementary data online, *eFigures S5* and *S6*).

### Falsification endpoint

**Figure 3:**
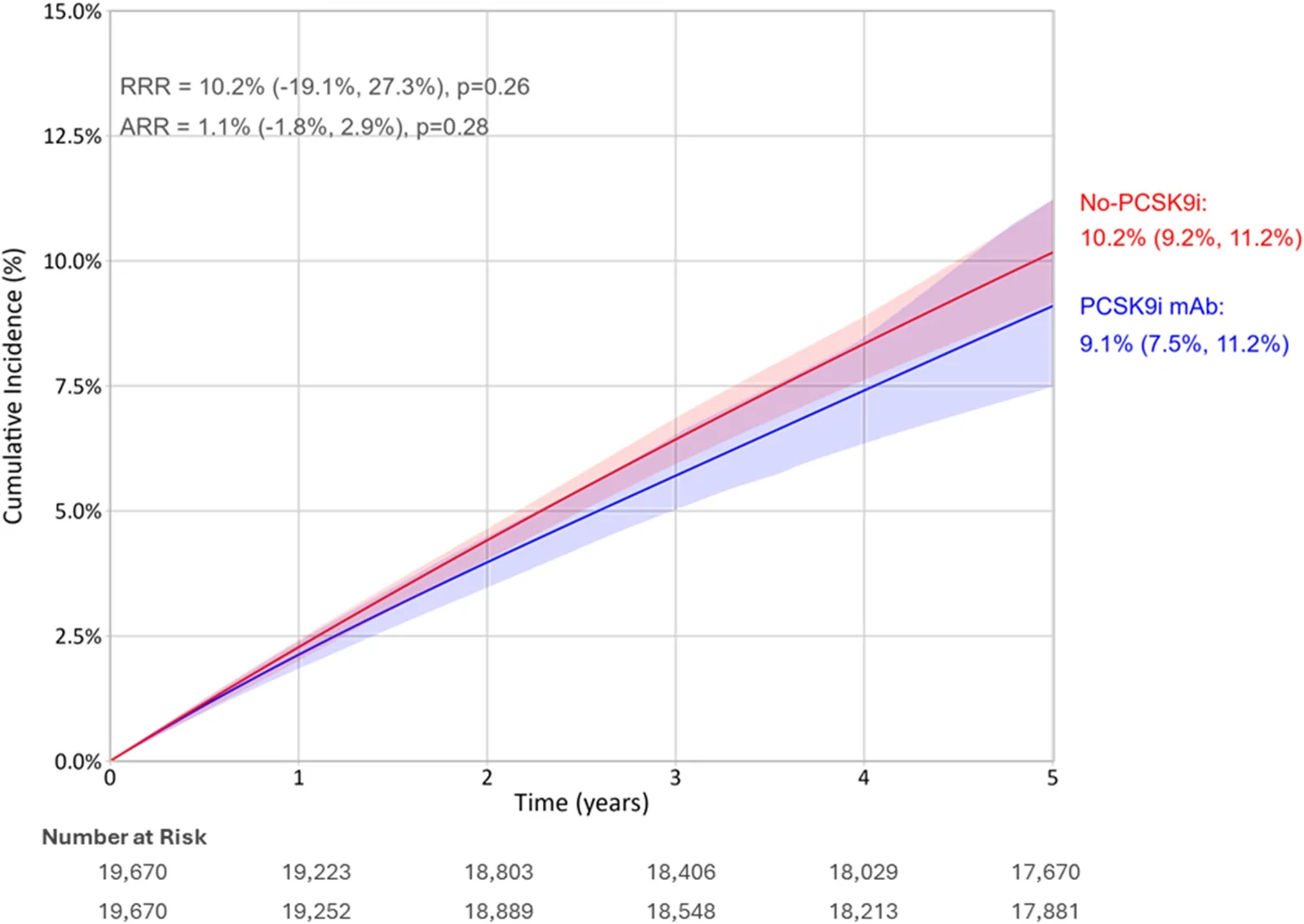
Falsification endpoint (incident cancer, major bleeding, or sepsis) for the study population—intention to treat analysis. ARR, absolute risk reduction; mAb, monoclonal antibody; PCSK9i, proprotein convertase subtilisin/kexin type 9 inhibitor; RRR, relative risk reduction. Numbers in parenthesis represent the 95% confidence intervals

At 5 years, the composite falsification endpoint occurred in 9.1% (95% CI 7.5%, 11.2%) of patients receiving PCSK9i mAb and 10.2% (95% CI 9.2%, 11.2%) of those without PCSK9i, corresponding to an RRR of 10.2% (95% CI −19.1%, 27.3%; *P* = .26) and an ARR of 1.1% (95% CI −1.8%, 2.9%; *P* = .28) (*Figure 3*). This non-significant difference supports the specificity of the primary findings and helps mitigate concerns about residual confounding.

## Discussion

The present analysis provides an estimate of the effectiveness of PCSK9i mAb therapy in usual clinical practice population, finding an almost 30% lower rate of ischaemic events (*Structured Graphical Abstract*). Notably, the study population represented patients with stable ASCVD without prior ischaemic events, and thus provides data on a large and important group of patients distinct from those in the completed randomized trials—but with a very high ischaemic event rate despite their apparent clinical stability.^14–19^ This population closely mirrors that enrolled in the recent VESALIUS-CV trial,^20^ which evaluated PCSK9i mAb therapy in a high-risk group without prior ischaemic events. In VESALIUS-CV, evolocumab reduced the risk of 3-point MACE (comprising coronary heart disease death, MI, or ischaemic stroke) by 25% [hazard ratio (HR) 0.75; 95% CI 0.65, 0.86], with consistent benefit across subgroups and endpoints. In contrast, ODYSSEY OUTCOMES randomized patients with a recent ACS, and FOURIER randomized patients with history of MI, non-haemorrhagic stroke, or symptomatic peripheral artery disease.^1,2^ Therefore, these current data provide insights about PCSK9i mAb that are complementary to the randomized trial data to date.

The estimated RRR for PCSK9i mAb was 30.9% with ITT analysis. These estimates were higher than the ∼20%–25% RRR from the randomized PCSK9i mAb trials. One explanation may be the longer follow-up duration here than in ODYSSEY OUTCOMES^1^ or FOURIER.^2^ Another explanation is that 38.6% of patients in this study were not on a statin, and 27.4% were not on any lipid-lowering therapy at all. ASCOT-LLA,^21^ CARDS,^22^ and JUPITER^23^ randomized high-risk populations without prior ischaemic events to statin vs placebo and found RRRs of 36%, 37%, and 44%, respectively, which are comparable to the present analysis. The results of our study, where the baseline LDL-C was 116.5 mg/dL, are also concordant with findings from the ODYSSEY OUTCOMES subgroup with baseline LDL-C > 100 mg/dL, in whom there was also a lower rate of mortality with PCSK9i mAb use.^1,3^ Statin naïve patients may have plaque that is particularly responsive to potent LDL-C reduction such as that achieved with PCSK9i mAb, and additionally those patients not treated with statins may have higher event rates. Patients in clinical practice have suboptimal rates of long-term statin adherence.^24,25^ Additionally, the current analyses were performed on patients actually receiving PCSK9i mAb (with confirmed filled prescriptions at the pharmacy) as opposed to the ITT analyses in randomized outcome trials of PCSK9i mAb.

Baseline statin use in our study was 61.4%, compared with 87% in VESALIUS-CV, where RRR was consistent regardless of statin therapy at baseline, a finding replicated in our study with the magnitude of relative benefit being consistent between the statin and no statin groups. The fact that only 61.4% of our cohort was receiving statin therapy at baseline reflects prior evidence from usual clinical practice and underscores a persistent gap in implementing guideline-directed lipid-lowering therapy. Multiple studies based on large, contemporary ASCVD populations in the USA have consistently reported statin use in the 50% to 70% range,^25–28^ with similar underutilization observed in Europe and other regions.^29^ These observations highlight therapeutic inertia, lack of awareness of the most recent lipid guidelines, and concerns by patients about side effects.

Extrapolating from the CTT data, the expected RRR over 5 years for the ITT analysis is 28.8% (95% CI 22.6%, 35.0%) vs 30.9% (95% CI 22.3%, 38.1%) from our study. The mean follow-up in the CTT meta-analysis was 5 years which is comparable to the 5-year follow-up of the current study. The findings from the present study were also concordant with VESALIUS-CV including percent LDL-C reduction (53.6% and 54.3%, respectively), baseline LDL-C (116.5 mg/dL and 122 mg/dL, respectively), and the approximate point of separation in Kaplan–Meier curves at around 9 months in both.

Although advanced methodologies for comparative evaluation were employed in this analysis, the possibility of residual confounding and bias inherent to non-randomized study designs cannot be entirely excluded. Potential sources include indication bias and socioeconomic factors. However, because this study used US data from the Optum Research Database—comprising commercially insured individuals and Medicare Advantage enrolees—the population is relatively homogeneous with respect to insurance coverage and access to care, mitigating some socioeconomic disparities compared with uninsured or publicly insured populations.

Additionally, there was no treatment effect on the composite falsification endpoint of incident cancer, hospitalized major bleeding, or hospitalized sepsis, providing reassurance that PCSK9i mAb use was not a proxy for a healthy user effect. Furthermore, the lower rate of ischaemic events seen here matched nicely with what would have been predicted by the CTT meta-analysis based on the magnitude of LDL-C reduction. Statins and other lipid-lowering therapies were not optimally used, but this phenomenon has been seen in several registries^24–29^ and reflects real-world practice.^30,31^ A certain proportion were likely statin intolerant, though that information was not captured. Events were not adjudicated, though it is unlikely that any misclassification of events would have differed between study arms. Non-prescription medications such as aspirin could not be captured. This study excluded a small proportion (∼0.4%) of patients on PCSK9i siRNA (inclisiran) at baseline. Secondary and primary prevention trials examining MACE for this agent are ongoing. Another limitation was that cardiovascular death was not separately analysed, though results from VESALIUS-CV in a similar event-free high-risk population found consistent effects of the PCSK9i mAb evolocumab on cardiovascular death (HR 0.79; 95% CI 0.64, 0.98) and all-cause death (HR 0.80; 95% CI 0.70, 0.91).

## Conclusion

In conclusion, in patients with ASCVD but without prior ischaemic events, PCSK9i mAb therapy compared with no treatment with PCSK9i was associated with a lower rate of major cardiovascular events, including mortality. The substantial magnitude of the benefit observed, with approximately 30% RRR in this population representing usual clinical practice with longer follow-up duration and less statin use than in the randomized PCSK9i trials, matched the CTT meta-analysis data closely, with an observed LDL-C reduction of nearly 39.8 mg/dL (1.0 mmol/L; ITT analysis) from baseline, which would be predicted to result in a ∼30% RRR in clinical events. Over one quarter of the patients not on PCSK9i therapy developed an ischaemic event over 5 years, justifying more intensive medical treatment. This analysis provides reassurance that PCSK9i mAb therapy appears to reduce cardiovascular events in a real-world population at least as much as observed in the randomized PCSK9i mAb trials and additionally demonstrates the utility of contemporary comparative evaluation methods to address clinical questions and provide information complementary to that obtained from randomized trials.

## Supplementary Material

ehag176_Supplementary_Data

## Declarations

### Data Availability

The underlying data contains proprietary elements that cannot be broadly disclosed or made publicly available. However, these data can be made available to a third party via the corresponding author upon reasonable request, assuming data security and privacy protocols are in place, and that the third party has executed the standard licence agreements.
