# Supplementary material for: PCSK9 inhibitor treatment and outcomes in patients with atherosclerotic cardiovascular disease but without prior ischaemic events: an observational study
Source: Eur Heart J. 2026 Apr 16;47(31):4277–86. doi: 10.1093/eurheartj/ehag176 (PMC13472681; doi:10.1093/eurheartj/ehag176)
Supplement: Supplementary file 1 [file ehag176_supplementary_data.pdf]

Supplement

eTable S1. Selection of Patients Initiating PCSK9i mAb.

Starting N	Selection Criteria	Remaining N	Excluded
Entire Database* 34,778,388	Initiation of PCSK9i mAb during January 2016 to December 2022	58,585	NA
58,585	Continuous enrolment in health plan during 1-year pre-index†	33,040	25,545
33,040	Enrolment in health plan at day-1 post-index	33,030	10
33,030	Age ≥ 18 years as of index year	33,026	4
33,026	Known demographic information	33,013	13
33,013	No ACS event during 1-year pre index	30,288	2,725
30,288	Evidence of ASCVD pre-index	24,585	5,703
24,585	No evidence of ischemic events pre-index	15,091	9,494
15,091	Matched to the comparator group and with non-missing baseline LDL-C	6,545	8,546

* Includes those in the Optum Research Database (ORD) over the age of 18 with at least 1 day of medical and pharmacy coverage during the identification period; † Index corresponds to therapy initiation (time zero).

Abbreviations: ACS, acute coronary syndromes; ASCVD, atherosclerotic cardiovascular disease; LDL-C, low-density lipoprotein cholesterol; PCSK9i mAb, proprotein convertase subtilisin/kexin type 9 inhibitor monoclonal antibody.

eTable S2. Selection of PCSK9i Non-Initiators.

Starting N	Selection Criteria	Remaining N	Excluded
Entire Database* 34,778,388	ASCVD during January 2015 to December 2022, without ischemic events either concurrent with or prior to earliest indication of ASCVD in the data.	3,712,898	NA
3,712,898	Random selection of patients and index fulfilling the eligibility criteria.	521,518	3,191,380
521,518	Initial 1:5 matching. All baseline variables except baseline LDL-C used in the propensity score.	66,470	455,048
66,470	Final 1:2 matching with non-missing baseline LDL-C. All baseline variables and baseline LDL-C used in the propensity score.	13,125	53,345

*Includes those in the Optum Research Database (ORD) over the age of 18 with at least 1 day of medical and pharmacy coverage during the identification period; †Index corresponds to therapy initiation (time zero).

Abbreviations: ASCVD, atherosclerotic cardiovascular disease; LDL-C, low-density lipoprotein cholesterol.

eTable S3. Baseline Characteristics of the Expanded Population.

	**PCSK9i mAb (n = 13,089)**	**No PCSK9i (n = 23,671)**	**Expanded Population (n = 36,760)**
Demographics, %			
Age, years			
Mean (SD)	68.2 (9.1)	68.7 (10.7)	68.6 (10.3)
18-44	1.3	1.6	1.5
45-64	29.5	29.9	29.8
65+	69.2	68.5	68.7
Male sex	50.5	50.2	50.3
Insurance			
Medicare Advantage*	69.1	70.3	69.9
Commercial	30.9	29.7	30.1
Atherosclerotic Cardiovascular Disease Characteristics, %			
Coronary Conditions	77.5	76.8	77.1
Stable Angina	75.4	74.4	74.7
Other IHD	18.0	17.6	17.7
Prior Revascularization	28.0	27.0	27.3
PCI	17.9	17.4	17.5
CABG	13.3	12.9	13.0
Ischaemic Cerebrovascular Disease	9.3	9.3	9.3
Peripheral Artery Disease	18.1	18.6	18.4
Lower extremities	3.7	3.8	3.8
Aorta	4.2	4.4	4.3
Other†	10.2	10.4	10.3
Other Comorbidities, %			
Diabetes	35.3	35.4	35.3
No insulin	23.2	23.4	23.3
On insulin	12.1	12.0	12.0
Hypertension	78.9	79.0	79.0
Liver disease	4.7	4.8	4.8
COPD	10.3	10.2	10.2
Heart failure	9.2	8.8	8.9
Chronic Kidney Disease	10.5	10.2	10.3

Stage III	9.0	8.7	8.8
Stage IV‡	1.5	1.5	1.5
Atrial fibrillation	10.0	10.0	10.0
Tobacco use	18.8	18.6	18.6
Cancer	11.7	11.3	11.4
Diagnosis	8.7	8.8	8.8
Medication	5.0	4.8	4.9
LLT use during 1-year prior to Index, %			
Any statin use	58.8	59.7	59.4
Low-Intensity	5.6	5.9	5.8
Moderate-Intensity	23.1	24.4	24.0
High-Intensity	30.1	29.4	29.6
Ezetimibe	32.3	32.7	32.6
Ezetimibe without statin	11.4	11.3	11.3
Icosapent ethyl	2.8	2.9	2.8
Bempedoic acid	0.4	0.4	0.4
Any statin, ezetimibe or bempedoic acid	70.3	71.1	70.8
Other CV SOC during 1-year prior to Index, %			
Beta-blockers	54.6	53.9	54.1
ACEi / ARB	58.2	58.2	58.2
Antiplatelets§	24.6	24.2	24.3
Anticoagulants	11.0	10.9	10.9
LDL-C Measures			
N, non-missing (%)	6,545 (50.0)	13,125 (55.4)	19,670 (53.5)
Mean (SD), mmol/L	3.1 (1.1)	3.0 (1.1)	3.1 (1.1)
Mean (SD), mg/dL	118.8 (41.7)	117.7 (43.3)	118.0 (43.6)
Median, mmol/L	3.0	2.9	2.9
Median, mg/dL	116.8	113.0	114.0

Abbreviations: ACEi, angiotensin-converting-enzyme inhibitor; ARB, Angiotensin receptor blocker; ASCVD, atherosclerotic cardiovascular disease; CABG, coronary artery bypass graft; COPD, chronic obstructive pulmonary disease; CV, cardiovascular; IHD, ischemic heart disease; LDL-C, low-density lipoprotein cholesterol; LLT, lipid-lowering therapy; PCI, percutaneous coronary intervention; PCSK9i mAb, proprotein convertase subtilisin/kexin type 9 inhibitor monoclonal antibody; SD, standard deviation; SOC, standard of care.

* Represents health plan in the US offered by a private company that contracts with Medicare to provide patients with hospital and medical insurance benefits.

† Includes any type of disease that is noncoronary, non-cerebrovascular, or does not involve the lower extremities or aorta.

‡ Includes dialysis or renal transplant.

§ Includes clopidogrel, ticagrelor, and prasugrel; 98.8% use in this category was for these agents. Additional agents were anagrelide, cilostazol, dipyridamole (including aspirin combo), pentoxifylline, and vorapaxar sulphate.

Percentages for ASCVD characteristics may exceed 100% because many patients have disease in multiple vascular beds.

eTable S4. Individual Endpoints for the Study Population – Intention-to-Treat Analysis.

	**PCSK9i mAb: Event Rate at 5-years (%)**	**No PCSK9i: Event Rate at 5-years (%)**	**RRR (%): Ref. No PCSK9i**	**ARR (%): Ref. No PCSK9i**
Nonfatal MI	5.2 (4.3, 6.3)	7.3 (6.6, 8.1)	28.3 (12.0, 40.6)<br>p<0.0001	2.1 (0.8, 3.1)<br>p<0.0001
Nonfatal Ischemic Stroke	4.0 (3.1, 4.7)	5.4 (4.6, 6.3)	26.4 (6.5, 45.7)<br>p=0.02	1.5 (0.3, 2.7)<br>p=0.02
All-Cause Mortality	8.7 (7.5, 9.8)	12.2 (11.2, 13.0)	28.5 (16.6, 39.1)<br>p<0.0001	3.5 (2.0, 4.9)<br>p<0.0001

Abbreviations: ARR, absolute risk reduction; MI, myocardial infarction; PCSK9i mAb, proprotein convertase subtilisin/kexin type 9 inhibitor monoclonal antibody; RRR, relative risk reduction.

Numbers in parenthesis represent the 95% confidence intervals. Rates for individual endpoints will not sum to the composite rate because, in the component-level analysis, occurrences of other endpoints are treated as censoring events rather than as competing risks.

eFigure S1. Random Index Selection for PCSK9i Non-Initiators.

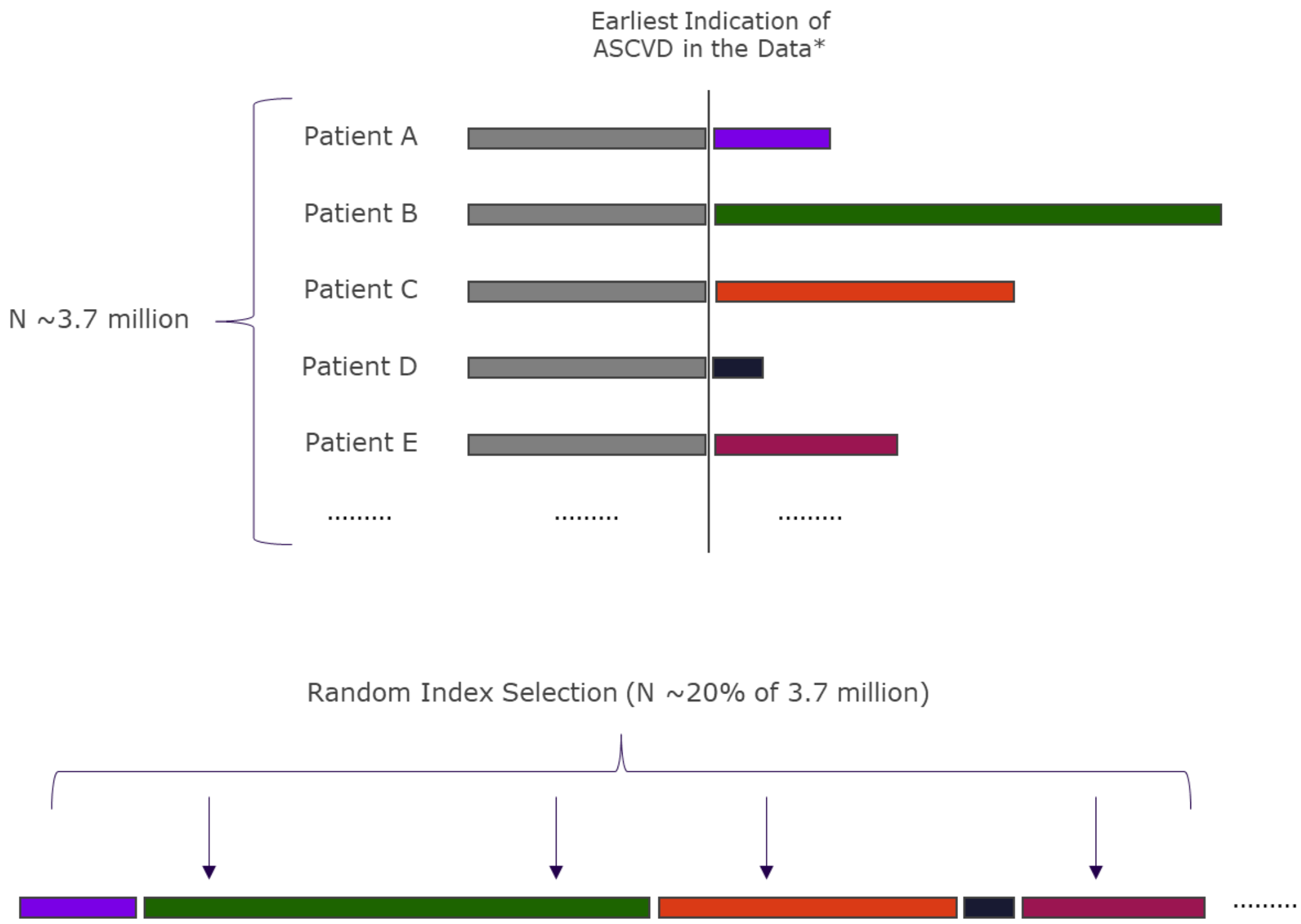

Abbreviations: ASCVD, atherosclerotic cardiovascular disease

* Patients with ischemic events concurrent or prior to this point were excluded.

eFigure S2. Cumulative Incidence of the Primary Composite Endpoint via 1 – KM Estimate.

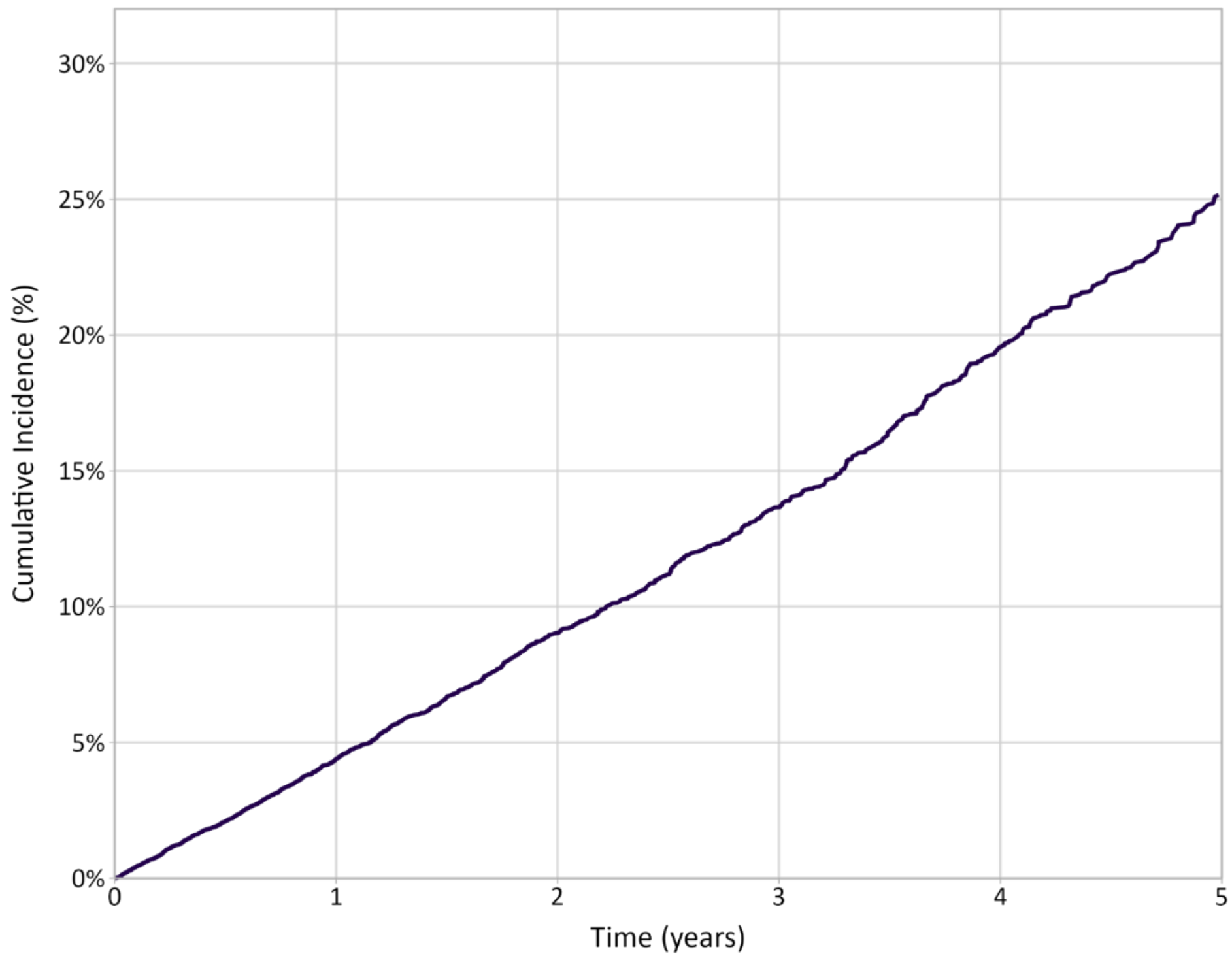

	0	1	2	3	4	5
**Number at Risk**	19,670	10,974	5,999	3,049	1,494	676
**Cumulative number of events**	0	749	1,183	1,419	1,567	1,630
**Cumulative number of censored patients**	0	8,392	13,312	16,298	17,935	18,803

Abbreviations: KM, Kaplan-Meier.

Unadjusted event rates over time for the primary composite endpoint for the overall study population of 19,670 individuals. The curve represents the cumulative incidence via 1 – KM (Kaplan-Meier), with the reasons for censoring being end of enrolment from the health plan, or end of study period. The relatively high rate of censoring by year 5 is driven mainly by disenrollment from the health plan, which is well-known in this database.[4] The absolute number at risk at year 5 is 676.

eFigure S3. Primary Composite Endpoint for the Expanded Population – Intention-to-Treat Analysis.

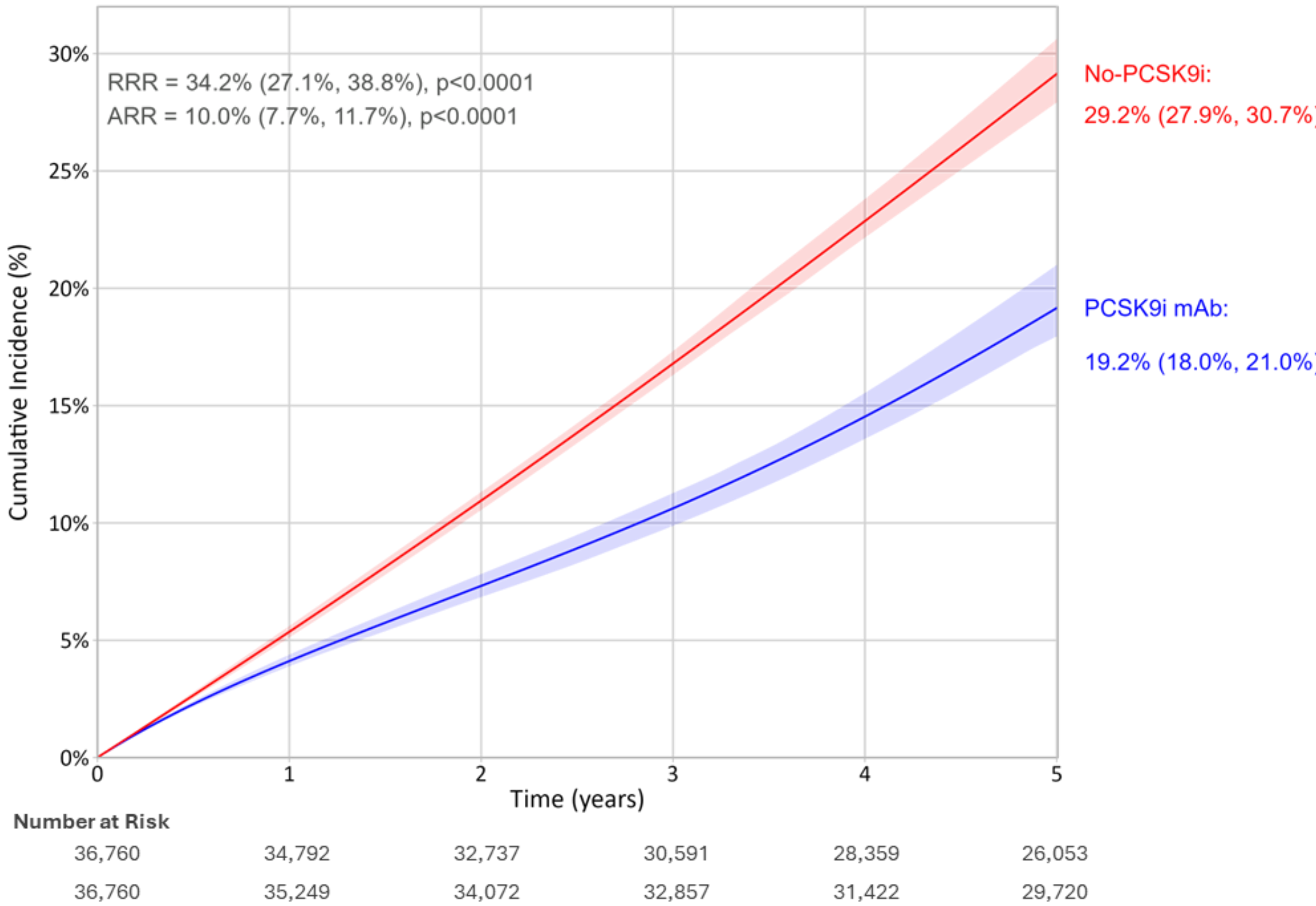

Abbreviations: ARR, absolute risk reduction; PCSK9i mAb, proprotein convertase subtilisin/kexin type 9 inhibitor monoclonal antibody; RRR, relative risk reduction.

Numbers in parenthesis represent the 95% confidence intervals.

eFigure S4: Primary Composite Endpoint for With and Without Baseline Statin Subgroup -– Intention-to-Treat Analysis**.**

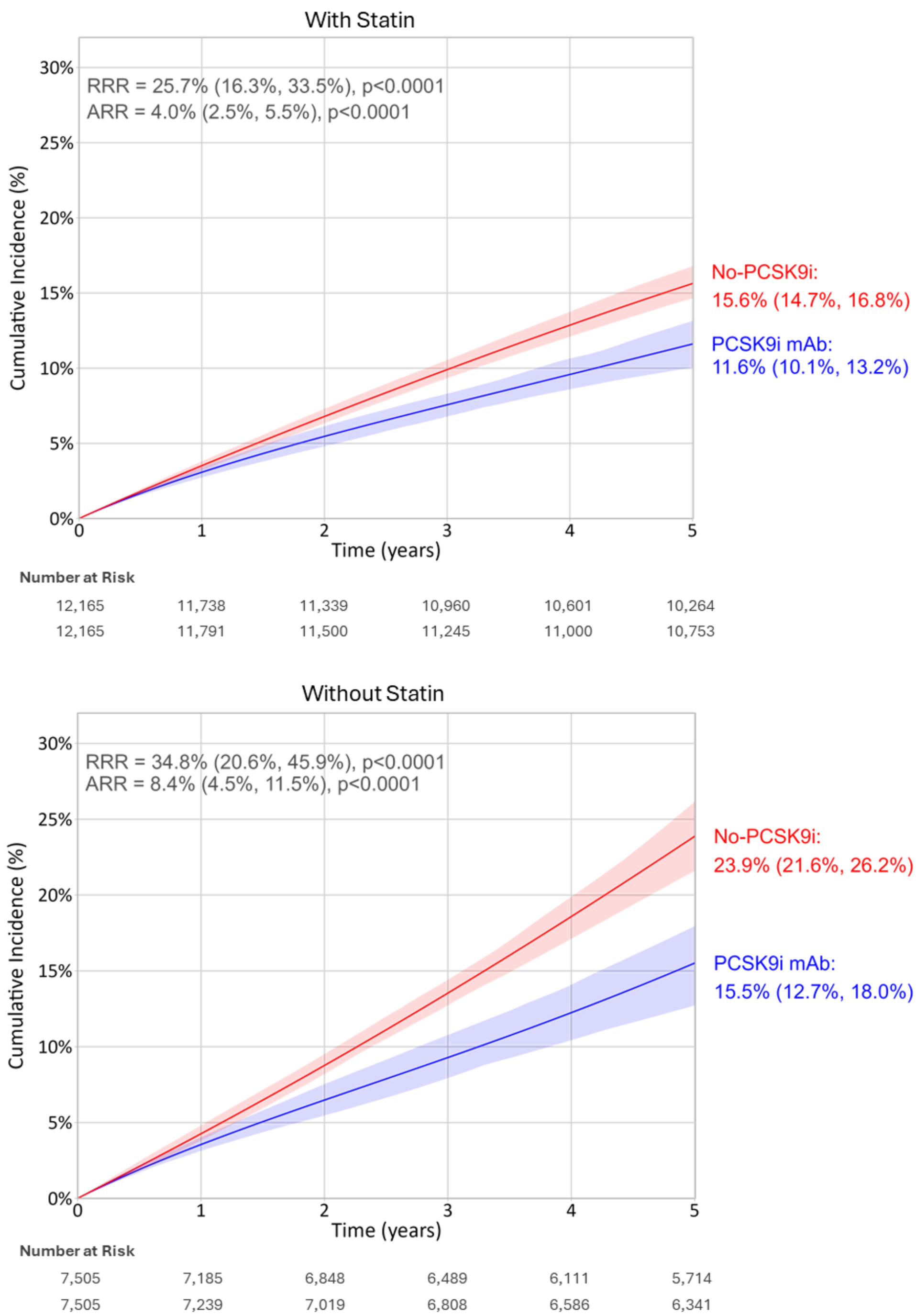

Abbreviations: ARR, absolute risk reduction; PCSK9i mAb, proprotein convertase subtilisin/kexin type 9 inhibitor monoclonal antibody; RRR, relative risk reduction.

Numbers in parenthesis represent the 95% confidence intervals. The difference in RRR between patients with and without baseline statin was not statistically significant (p=0.42).

eFigure S5: Primary Composite Endpoint for the Study Population – Per-Protocol Analysis.

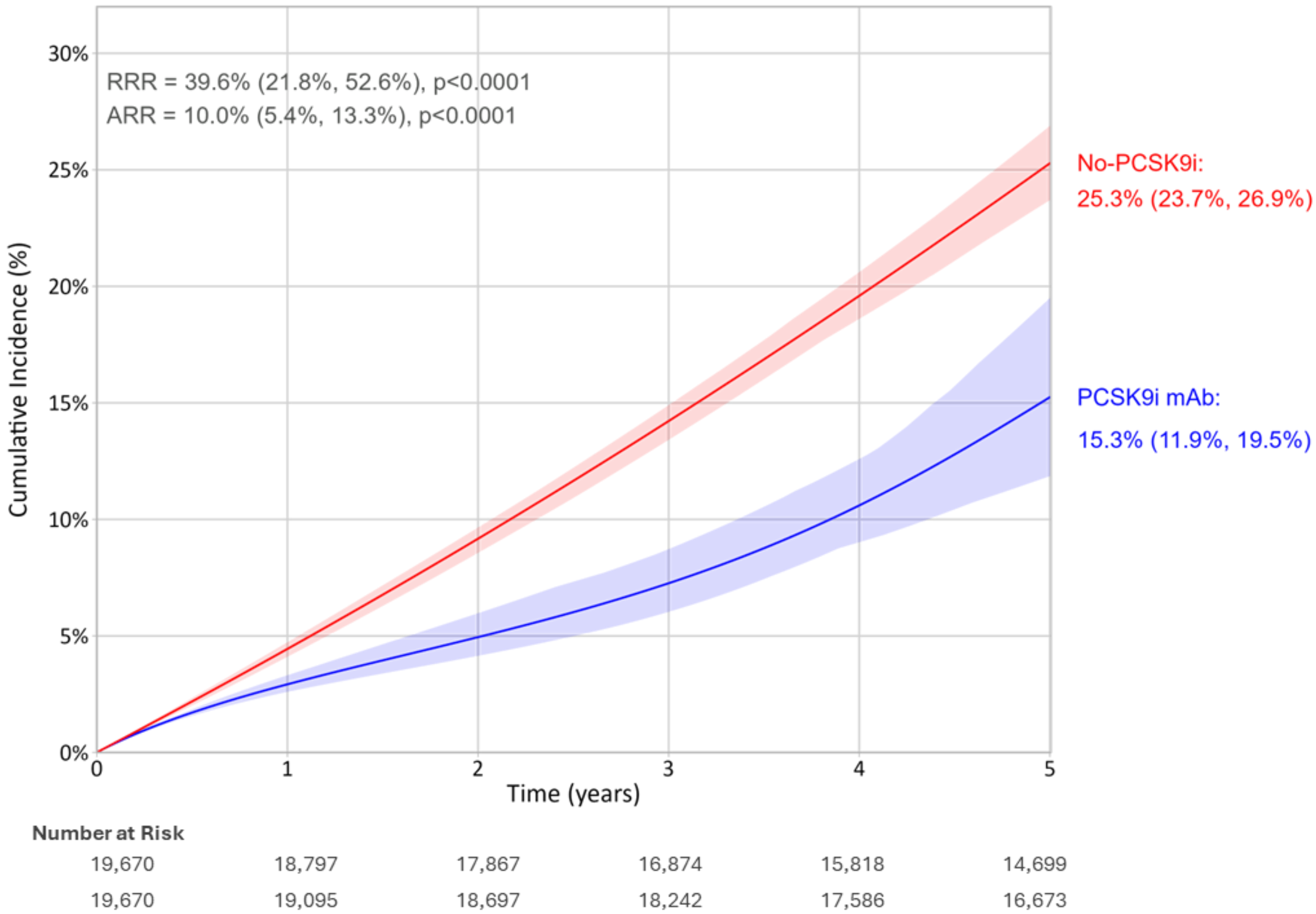

Abbreviations: ARR, absolute risk reduction; PCSK9i mAb, proprotein convertase subtilisin/kexin type 9 inhibitor monoclonal antibody; RRR, relative risk reduction.

Numbers in parenthesis represent the 95% confidence intervals.

eFigure S6: Mean Low-Density Lipoprotein Cholesterol Over Time in the Study Population.

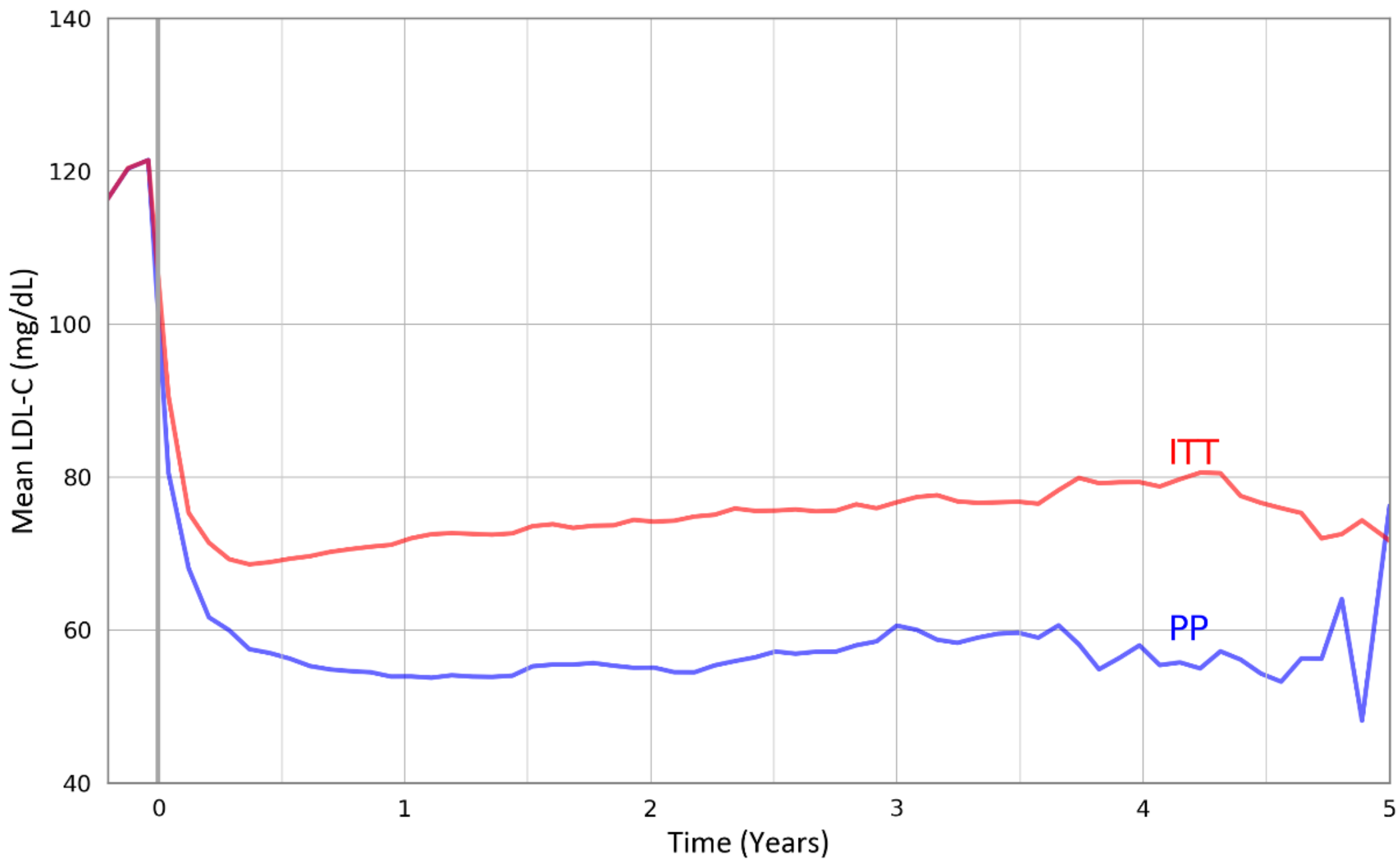

Abbreviations: ITT, intention-to-treat; LDL-C, low-density lipoprotein cholesterol; PP, per-protocol

eMethods S1: PubMed Search for Observational Evidence on PCSK9 Inhibitor Effectiveness

To identify observational evidence on the effectiveness of proprotein convertase subtilisin/kexin type 9 inhibitors (PCSK9i) for hard cardiovascular outcomes (e.g., myocardial infarction, stroke, or mortality), we conducted a targeted PubMed search using the following terms:

- ("PCSK9 inhibitor" OR alirocumab OR evolocumab) AND (real-world OR observational OR registry OR "clinical practice") AND (ASCVD OR "atherosclerotic cardiovascular disease") AND (outcomes OR endpoints OR effectiveness OR "cardiovascular events" OR "coronary events" OR "cerebrovascular events" OR MACE OR mortality)

Variants of this query were also tested (e.g., omitting ASCVD or substituting "events" with "outcomes" or "endpoints"). All retrieved records were manually reviewed to confirm inclusion of major cardiovascular events or mortality as endpoints.

Results: The search identified limited observational evidence evaluating PCSK9i effectiveness for hard outcomes. Only one large-scale study reported robust findings (Jin et al., 2025; congress abstract), while two additional peer-reviewed studies were underpowered.

Summary of Identified Studies

Jin et al. 2025[1] (Congress Abstract)

- Population: 88,713 evolocumab initiators with ASCVD (continued therapy) vs. 25,075 early discontinuers.
- Endpoint: Composite of myocardial infarction, stroke, and coronary revascularization.
- Follow-up: 4 years.
- Findings: 21% lower risk (rate ratio = 0.79; 95% CI: 0.73–0.86) in treated group.
- Status: Abstract only; full text unavailable.

Zhang et al. 2024[2] (Peer-Reviewed)

- Population: 206 evolocumab-treated acute ischemic stroke patients vs. 322 SOC controls (propensity matched).
- Endpoint: Composite major adverse cardiovascular events (MACE; coronary/cerebrovascular events and event-related deaths).
- Follow-up: 12 months.
- Findings: No significant reduction in composite MACE (HR = 0.68; 95% CI: 0.44–1.06).

Kim et al. 2023[3] (Peer-Reviewed)

- Population: 91 PCSK9i initiators vs. 840 matched controls with ASCVD or familial hypercholesterolemia.
- Endpoint: Composite of all-cause mortality, major cardiovascular events, and ischemic stroke.
- Follow-up: 178 days (median).
- Findings: No significant differences (all $p > 0.05$).

Interpretation: Observational studies assessing PCSK9i effectiveness for hard cardiovascular outcomes remain scarce. Available evidence is either unpublished in full-text form or underpowered.

eMethods S2. Development of the Study Population.

The entire Optum Research Database contains over 34 million patients that have at least a day of medical and pharmacy coverage during the identification period. From this database, patients who initiated PCSK9i mAb between January 2016 and December 2022 were identified, narrowing the sample to 58,585. Further eligibility reduced this cohort: continuous enrolment in a health plan during the 1-year pre-index period (33,040 patients remaining), enrolment on day 1 post-index (33,030 remaining), and age ≥18 years at the index year (33,026 remaining). Exclusions accounted for missing demographic information (33,013 remaining) and pre-index ACS events (2,725 excluded), leaving 30,288 patients. Among these, 24,585 had evidence of ASCVD pre-index. Excluding those with pre-index ischemic events resulted in 15,091 patients. Finally, matching with comparator population who have non-missing baseline LDL-C measurements eliminated 8,546 patients, yielding a final analytic cohort of 6,545 patients initiating PCSK9i mAb.

The comparator population was developed as follows. From the initial available pool of over 34 million individuals, 3,712,898 were identified with ASCVD from January 2015 to December 2022, excluding those with concurrent or prior ischemic events. Conceptually, any point after this and before a potential ischemic event can be assigned as an index (time zero), in spirit of a target trial emulation (point where this patient is eligible for a hypothetical trial). In order to make a pragmatic and valid selection of a subset of patients and index dates, we randomly selected index dates proportional to the amount of eligible follow-up per schematic below and proceeded to evaluate whether each one of the selected index met other eligibility criteria summarized in eTable 1. This resulted in 521,518 patients being retained. After the initial 1:5 matching (all variables except LDL-C in propensity score) 66,470 patients were retained. After subsequent matching with 1:2 ratio (only including those with non-missing baseline LDL-C measurements, and including baseline LDL-C in the propensity score), the final comparator group of 13,125 was retained.

A broader population including those with missing LDL-C baseline measurements was also developed for supplementary analysis. The missing baseline LDL-C values were first imputed based on gamma regression with the model learned from the above developed population with non-missing LDL-C baseline measurements. Then a 1:2 propensity score matching was performed. For patients with missing baseline LDL-C measurements, 7,276 PCSK9i mAb initiators and 13,262 No PCSK9i users were obtained. Eventually, it yielded a final analytic cohort of 13,089 patients initiating PCSK9i mAb, and a final comparator group of 23,671 patients.

eMethods S3. Comparative Evaluation via Parametric G-Formula.

Modern statistical methods for comparative evaluation with observational data have been described comprehensively in the textbooks by Pearl et al. and Hernan et al.[5,6] Because these methods have been relatively less utilized compared to propensity score matching, we provide highlights in this section.

Estimation of the effect of a therapy with non-randomized data are possible under the key identifying assumptions of consistency, positivity, and conditional exchangeability. Briefly, the consistency assumption means that treatment received by patients corresponds to one of the defined interventions of interest (treat with PCSK9i mAb, do not treat with PCSK9i), and that outcomes are accurately ascertained. The positivity assumption means there are no profiles in the database where the physician in the real-world would never or always assign a PCSK9i mAb to these patients. In other words, the probability of receiving treatment must be between 0% and 100% within each patient profile in the analysis dataset. Finally, conditional exchangeability means, if in the database one encounters two identical profiles, with one receiving PCSK9i mAb and another one not, then they are effectively randomized. An alternative way of stating this is that all relevant confounders were available in the confounder set.

Statistical approaches for unbiased estimation of treatment effect are possible if the above identification assumptions hold. The key options for statistical estimation are inverse probability weighting, marginal structural models, G-formula, and G-estimation. With high-dimensional data (i.e. a patient is characterized by a relatively large number characteristics), parametric models are needed, where G-formula is most amenable. The parametric version for G-formula is referred to as the Parametric G-Formula. Inference with the Parametric G-formula relies on the additional assumption of a correctly specified parametric model. For details see Hernan et al.[6] The parametric model underlying our analysis was a discrete-time logistic model,[7] which modelled the hazard over time, with covariates representing the confounder set (all baseline characteristics), an indicator for treatment variable (PCSK9i mAb), and flexibility to model time-varying hazard via a spline function. Estimation of the discrete-time logistic model for hazard utilized the generalized linear model (GLM) package in R programming language.[8] The estimated discrete-time hazard model was used in the Parametric G-Formula to yield event rate curves over time.

Confidence intervals were obtained by bootstrapping, where we repeatedly resampled from the source target population with the sample size in each bootstrap iteration remaining the same. We refitted the parametric models and re-estimated the event rate curves over time for each bootstrap iteration. The point estimates we report represent the median of bootstrap iterations, while the 95% confidence intervals represent the 2.5th and 97.5th percentiles of the bootstrap iterations.

eMethods S4. Estimation of Reduction in LDL-C with PCSK9i mAb.

We estimated LDL-C reduction in patients who initiated PCSK9i mAb and with available (non-imputed) LDL-C at baseline and follow-up. First, we identified patients who initiated PCSK9i mAb and with available (non-imputed) LDL-C at baseline. At the point of this baseline LDL-C measurement, we also recorded the background LLTs the patient was receiving (e.g., a statin and/or ezetimibe; for statin we also recorded the type and dose). From this set we retained those who had at least one LDL-C measurement during the follow-up that was concurrent with a PCSK9i mAb. At the point of these LDL-C measurements we also recorded the background LLTs the patient was receiving.

Because the background LLTs can change from baseline to follow-up for a given patient, eliminating bias in the estimation of reduction in LDL-C with PCSK9i mAb requires an adjustment such that other background LLTs remain identical during baseline and follow-up. Let L_0 denote the baseline LDL-C for a given patient and L_j' denote the jth available follow-up LDL-C measurement for this patient (we use the prime superscript to denote an unadjusted estimate). Let k denote a specific background LLT (e.g. atorvastatin 10 mg), I_{0k} denote an indicator flag for presence this agent at baseline LDL-C measurement, and I_{jk} denote the indicator flag for this agent at the follow-up LDL-C measurement j. We define an adjusted LDL-C at follow-up as:

$$L_j = L_j' \frac{(1 - e_1 I_{01}) \times \cdots \times (1 - e_K I_{0K})}{(1 - e_1 I_{j1}) \times \cdots \times (1 - e_K I_{jK})} \qquad 1$$

Where e_k represents the estimated efficacy in terms of percent LDL-C reduction for agent k. For example, $e = 0.414$ for atorvastatin 20 mg. For statins and ezetimibe this information is well-established, and we obtained it from Cannon et al.[9] As an example, if the patient was on atorvastatin 20 mg and ezetimibe at baseline and on atorvastatin 10 mg only at follow-up, then this calculation will yield:

$$L_j = L_j' \frac{(1 - 0.414) \times (1 - 0.227)}{(1 - 0.355)} = L_j' * 0.70 \qquad 2$$

In the above illustration the observed LDL-C at follow-up will be reduced by 30% to align with the more intensive background therapy at baseline. The absolute reduction in LDL-C with PCSK9i mAb for a given patient is $\Delta L = L_0 - \overline{L_J}$, where $\overline{L_J}$ is the mean across follow-up measurements. The mean of ΔL across all patients yielded the mean reduction in LDL-C with PCSK9i mAb.

References

1. Jin R, Hurwitz K, Kent S, Jackman L, Dhalwani N, Accortt N, et al. Real-World Effectiveness of Evolocumab in Reducing Major Adverse Cardiovascular Events in Patients with Atherosclerotic Cardiovascular Disease. Circulation. 2025;152(Suppl_3):4363106. doi:10.1161/circ.152.suppl_3.4363106.

2. Zhang T, Zhang Y, Yang Y, Liao H, Li X, Liu R, et al. Real-world Effectiveness and Safety of Evolocumab in Very High-risk Atherosclerotic Cardiovascular Disease Patients with Acute Ischemic Stroke. J Thromb Thrombolysis. 2024 Feb;57(2):302-311. doi: 10.1007/s11239-023-02925-4.

3. Kim OM, Givens TK, Tang EG, Schimmer JJ, Ramsey T, Boyd K, Delate T. Real-World Outcomes of Proprotein Convertase Subtilisin Kexin-9 Inhibitor Use. J Cardiovasc Pharmacol. 2023 May 1;81(5):339-347. doi:10.1097/FJC.0000000000001404.

4. Steen DL, Khan I, Andrade K, Koumas A, Giugliano RP. Event Rates and Risk Factors for Recurrent Cardiovascular Events and Mortality in a Contemporary Post Acute Coronary Syndrome Population Representing 239 234 Patients During 2005 to 2018 in the United States. J Am Heart Assoc. 2022 May 3;11(9):e022198. doi: 10.1161/JAHA.121.022198. Epub 2022 Apr 27. PMID: 35475346; PMCID: PMC9238606.

5. Pearl J. *Causality: Models, Reasoning, and Inference*. 2nd Edition. Cambridge University Press. 2009.

6. Hernán MA, Robins JM. *Causal Inference: What If*. Boca Raton: Chapman & Hall/CRC. 2020.

7. Tutz G, Schmid M. Modeling Discrete Time-to-Event Data. Springer. 2016.

8. R Core Team. R: A Language and Environment for Statistical Computing. 2025. https://www.R-project.org.

9. Cannon CP, Khan I, Klimchak AC, Reynolds MR, Sanchez RJ, Sasiela WJ. Simulation of Lipid-Lowering Therapy Intensification in a Population with Atherosclerotic Cardiovascular Disease. JAMA Cardiology. 2017;2(9):959. doi: 10.1001/jamacardio.2017.2289.
